# Personalization of Mobile Apps for Health Behavior Change: Protocol for a Cross-sectional Study

**DOI:** 10.2196/38603

**Published:** 2023-01-05

**Authors:** Laetitia Gosetto, Marta Pittavino, Gilles Falquet, Frederic Ehrler

**Affiliations:** 1 Centre Universitaire d'Informatique Geneva School of Economics and Management University of Geneva Carouge Switzerland; 2 Research Center for Statistics Geneva School of Economics and Management University of Geneva Geneve Switzerland; 3 Division of Medical Information Sciences University Hospitals of Geneva Geneve Switzerland

**Keywords:** mobile health, mHealth, personalization, mobile app, behavior change theory, gamification, functionalities

## Abstract

**Background:**

Mobile health apps have the potential to motivate people to adopt healthier behavior, but many fail to maintain this behavior over time. However, it has been suggested that long-term adherence can be improved by personalizing the proposed interventions. Based on the literature, we created a conceptual framework for selecting appropriate functionalities according to the user's profile.

**Objective:**

This cross-sectional study aims to investigate if the relationships linking functionalities and profiles proposed in our conceptual framework are confirmed by user preferences.

**Methods:**

A web-based questionnaire comprising several sections was developed to determine the mobile app functionalities most likely to promote healthier behavior. First, participants completed questionnaires to define the user profile (Big Five Inventory-10, Hexad Scale, and perception of the social norm using dimensions of the Theory of Planned Behavior). Second, participants were asked to select the 5 functionalities they considered to be the most relevant to motivate healthier behavior and to evaluate them on a score ranging from 0 to 100. We will perform logistic regressions with the selected functionalities as dependent variables and with the 3 profile scales as predictors to allow us to understand the effect of the participants’ scores on each of the 3 profile scales on the 5 selected functionalities. In addition, we will perform logistic ordinal regressions with the motivation score of the functionalities chosen as dependent variables and with scores of the 3 profile scales as predictors to determine whether the scores on the different profile scales predict the functionality score.

**Results:**

Data collection was conducted between July and December 2021. Analysis of responses began in January 2022, with the publication of results expected by the end of 2022.

**Conclusions:**

This study will allow us to validate our conceptual model by defining the preferred functionalities according to user profiles.

**International Registered Report Identifier (IRRID):**

RR1-10.2196/38603

## Introduction

### Background

Healthy lifestyle behaviors have increased the life expectancy of those who adopt them and help individuals to live not only longer but better [1]. More specifically, adopting a healthy diet, maintaining a healthy weight, quitting smoking, drinking alcohol in moderation, and regular exercise are 5 behaviors associated with lower mortality. An increasing number of health apps aiming to help people adopt better health behaviors are reaching the market annually, with over 35,000 health apps available in 2018 [2]. Smartphone apps offer new opportunities to adopt health-related behaviors by providing immediate access to information about one's health, reminders to take medication, or help track one's progress [3].

Several scales exist to measure the quality of these health-related mobile apps, such as the Mobile App Rating Scale [4] and the App Behavior Change Scale [5]. A common feature of these scales is to consider a mobile app's personalization as a quality factor. Indeed, personalization is an important aspect to consider when creating an app that enables behavior change. For example, it has been shown that messages tailored to the user tend to be read more, recalled more, attract more attention, be better remembered, be a topic of discussion with others, and be perceived as personally relevant compared to untailored messages [6].

### Development of a Mobile App Model for Behavior Change

Based on a previous literature review, we identified the personality traits more likely to adopt certain app functionalities [7]. These findings led to the development of a model indicating the type of features preferred according to a user profile. When designing a mobile app aiming at behavior change for health, designers can refer to our model as a guideline to know what functionalities they should privilege for their apps, given the profile of the intended users. For example, if a person is extroverted according to the Big Five, it will be relevant to privilege functionalities allowing comparison and cooperation between users [8].

Our model contains 17 functionalities presented in detail in Multimedia Appendix 1. For the user profile, we relied on the most common classification dimensions found in the literature, as follows: personality profiles [8-16], game preference [11,17,18], and perception of social norm [19] (Table 1). Gender and age are also important, and a recent review showed a difference in the type of functionality preferred according to gender, although no study in the review included individuals older than 31 years [17].

One of the most popular scales to measure personality is the Big Five, which defines the user’s personality according to the following 5 dimensions: openness, agreeableness, conscientiousness, neuroticism, and extraversion. Game preference was measured with the Hexad Scale model [18], which defines the user's gamer profile according to the following 6 dimensions: disruptor, achievers, free spirit, player, socializer, and philanthropist. For example, participants with the profile “player” are motivated by extrinsic rewards and will do anything to earn a reward within a system. This type of profile is interesting to consider for apps that use gamification, which is also a concept widely used nowadays to incite behavior change. We can define gamification as “the use of game design elements in non-game contexts” [21]. Indeed, gamification positively affects motivation, engagement, and enjoyment [22]. Finally, the perception of social norm is the “individual's perception that other individuals important to the respondent believe that the respondent should perform the behavior of interest” [23]. This perception can help or hinder the performance of the behavior, depending on how the user's entourage perceives it. Therefore, it is important to consider this factor, and, depending on this perception, different functionalities can be included.

**Table 1:** Profiles considered in our conceptual framework.

Profiles	Scale
Personality	Big Five
Game’s preferences	Hexad Scale [18]
Perception of social norms	Theory of Planned Behavior [20] Action

### Objectives

This study aims to validate our conceptual framework by investigating if the proposed relationships between the functionalities and profiles are reflected in the preferences of our target population in an experimental setting.

## Methods

### Ethics Approval

The University Ethics Commission has approved this study for ethical research at the University of Geneva (CUREG_2021-04-38).

### Study Design

We performed a cross-sectional study to address our aims. Participants responded to a web-based questionnaire to define their profile. Then, they were presented with a series of prototyped functionalities to be ranked according to their preferences to analyze if they corresponded to those defined in our conceptual framework. We chose to contextualize the functionalities of adopting healthy diet and fitness apps as these issues allow to target a generic public. Indeed, the desire to stay fit is a behavior that most adults want to adopt. To ensure data are completely anonymous, participants’ IP addresses were not collected. We tested the questionnaire for usability and technical issues with 5 participants. This web-based survey is in accordance with the Checklist for Reporting Results of Internet E-Surveys [24].

### Outcomes

The primary outcome is the preferred functionalities given the user profile.

Secondary outcomes are the feature preferences related to past or current use of mobile health (mHealth) apps, and the preference of functionalities according to the participant's state of motivation to change behavior.

### Study Population and Sample Size

The target population for this study included all individuals older than 18 years who understood French. We chose to conduct the questionnaire in French as this population was not necessarily fluent in English and comprised mainly native French speakers. An English language questionnaire would have introduced an element of bias as it might not have been correctly understood. Recruitment was conducted by posting messages on social networks (Facebook and Twitter) targeted at students at the University of Geneva, a young student population. The message indicated that we were seeking to recruit participants for a web-based study lasting 12 minutes as part of a research study conducted by the University of Geneva, with a focus on identifying user preferences based on their profile for a mobile app aimed at helping people get in shape. We also stated that the collected data remain completely anonymous.

For the calculation of the sample size, based on the hypothesis that altruistic people according to the Big Five prefer social networks [11,17], we used the multiple regression power calculation on R (R foundation for Statistical Computing), with the following measures: *u*=3; *f^2^*=0.07; *P*=.05; power=0.9; and variance=202.403. To estimate variance, we relied on a previous study [25] investigating the preference of users classified according to the Big Five on posters. More specifically, we looked at the variance of altruistic participants (n=46) according to the Big Five on the average ratings of a poster representing a social network promoting blood donation (score from 0 to 100). Thus, we obtained a sample size of 206.

### Procedure

Participants were asked to complete the web-based questionnaire developed using Qualtrics software (Qualtrics; Multimedia Appendix 2). First, they completed the consent form describing the purpose of the study and the procedure and informing them of their right to withdraw from the study. They were asked to confirm that they have read and understood the consent form and agree to have their responses used in our research and scientific publications. They can then access the rest of the questionnaire if they accepted these clauses. If not, they were informed that without their consent, we cannot collect their data and must terminate the survey. Next, participants were asked to answer demographic questions. In the case of a participant younger than 18 years, we explain that only those aged >18 years can participate and therefore we cannot continue with the questionnaire. Eligible participants continued to answer the questionnaire online where they had to (1) respond to scales to measure their profile, and then (2) look at the 17 features, select 5, and indicate on a score from 0 to 100 how much these features would motivate them to get back in shape.

### Measures and Measurement

#### Demographic Questions

Participants were asked to indicate their gender, age, occupation, and level of education.

#### Questions About Their Use of mHealth Apps

Participants were asked if they use mobile apps aiming at behavior change (such as to help them eat healthier or exercise) to find out if they were already familiar with mHealth apps and whether they already like certain functionalities. If so, we asked them to select which functionalities they used most often and which they never used. These questions allowed us to observe whether participants already familiar with mHealth prefer certain features, as well as whether they prefer the same features among the 17 proposed.

#### Profile Assessment

##### Big Five

To assess participants' personalities, we relied on the Big Five Inventory-10 scale in French, translated and validated by Courtois [26]. With Cronbach alpha coefficients ranging from .37 to .83, the internal scale reliability of the Big Five Inventory-10 is low. This is because Cronbach alpha is not designed to evaluate scales with a low number of items [26]. This scale is composed of 10 items, 2 items per Big Five dimension. Participants are asked to indicate on a 5-point Likert scale whether they strongly approve or strongly disapprove of statements about themselves. For example, “I see myself as someone who is reserved” or “I see myself as someone who is easily anxious”. The score for each dimension is calculated by adding the scores for the two statements concerning the dimension after reversing the items.

This scale was chosen because it has a factorial structure identical to that of the full version of the Big Five Inventory scale in French [26]. Therefore, it has the advantage of effectively measuring personality with a small number of items. As our protocol contains several scales, we preferred to choose the shortest valid versions to avoid participant fatigue with an excessively long questionnaire.

##### Gamer Profile

To identify participants' gamer profiles, we chose the Hexad Scale, created and validated by Tondello [18]. The internal scale reliability is good with Cronbach alpha coefficient for each dimension ranging from .70 to .89 [18]. This scale consists of 24 items, 4 per dimension. Users must rate how well each article describes them on a 7-point Likert scale. For example, there are items such as “I like competitions, where a prize can be won” or “Interacting with others is important to me.” Items are presented in a randomized manner, and the score is calculated by adding the scores for each dimension.

##### Perception of Social Norm

For the perception of social norm, we chose two items concerning this dimension of the Theory of Planned Behavior questionnaire of Ajzen [20]. We adapted the items to the context of our mobile app, which is to eat healthier and do more physical activity. Thus, the two items are as follows: “Most people who are important to me approve of the fact that I eat healthier and do more physical activity” and “Most people like me eat healthily and do physical activity.” Participants were asked to respond to these statements on a 7-point scale ranging from “agree” to “disagree.” The calculation was done by adding up the scores, with a high score indicating a heightened social norm perception.

#### Choice of Functionalities

##### Presentation of the Functionalities

**Figure 1:**
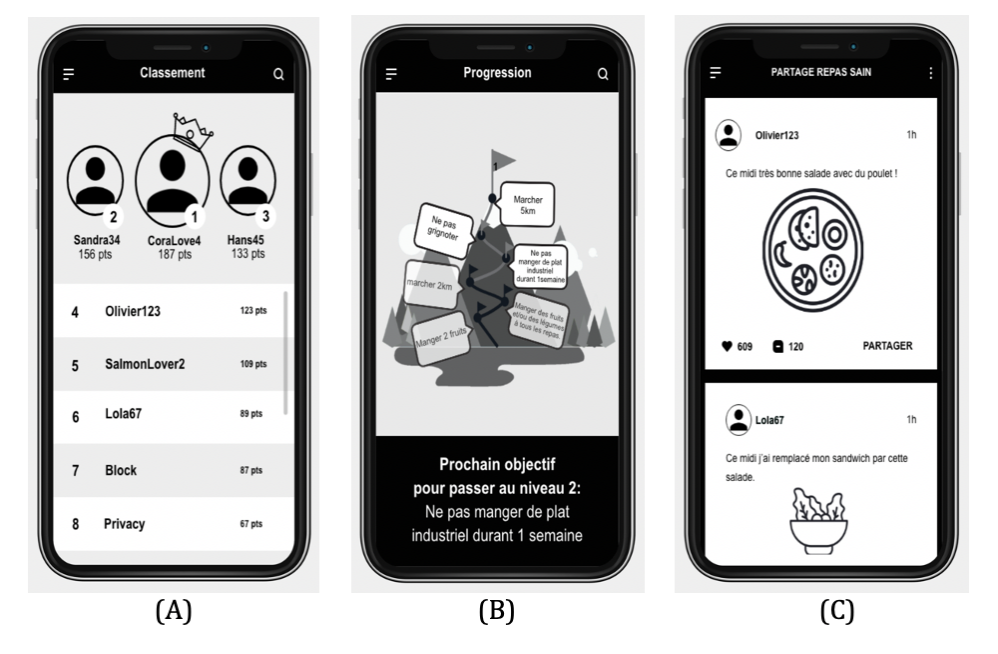
Example of screenshots of the prototype app, including the (A) functionality competition, (B) functionality level and progression, and (C) functionality social network.

From the literature, we identified 17 functionalities commonly proposed in behavior change apps. We then created a prototype for each of these functionalities. All functionalities and their definition are presented in Multimedia Appendix 1. We chose a visual design as neutral as possible for the prototypes (ie, in black and white with no images, only icons). This aims to minimize the bias due to design preference (ie, Figure 1). The 17 prototype screenshots were presented randomly to the participants to avoid a primacy or recency effect. During the study, participants discovered every functionality, one by one, by its representation in an image and accompanied by a short description. Then, they chose the 5 functionalities they considered to be the most motivating to stay fit.

##### Explanation of Choice

For each functionality selected, participants were asked to indicate how much that functionality would motivate them to adopt healthier behavior on a scale of 0 to 100. Then, they were asked why they chose these functionalities. Excluded functionalities will default to a score of 0.

### Analysis

#### Overview

We will exclude incomplete questionnaires and analyze only questionnaires that have been completed entirely.

Demographic characteristics of all participants will be presented using descriptive statistics (mean, standard deviations, or frequencies and range) in a table. A table will also provide responses about their use of mobile apps for health.

#### Quantitative Data

##### Primary Outcome

We will perform logistic regression with the functionalities as dependent variables and with scores of the 3 profile scales as predictors. This analysis will allow us to understand the effect of the participants’ scores on each of the 3 scales (Big Five Inventory-10 scale in French, Hexad Scale, and perception of the social norm) on the 5 selected functionalities. By performing a logistic regression for each feature, it will be possible to determine whether the scores on the different scales predict the selection of the functionality.

In addition, we will perform a logistic ordinal regression with the motivation score of the functionalities chosen as dependent variables and with scores of the 3 profile scales as predictors. By performing this regression for each functionality motivation score, it will be possible to determine whether the scores on the different scales predict the functionality score.

##### Secondary Outcome

To test whether there is a difference in functionality selection by age or gender, we will run logistic regressions with the choice of the functionality as the dependent variable and age or gender as the independent variables. In addition, we will perform an ordinal regression with the motivation score of the functionalities as the dependent variable and age or gender as the independent variable. There will be one regression per feature.

To test whether participants indicated that they preferred functionalities that are the same as the ones already used in their current mHealth app, we will run simple regressions with the feature they already use as the independent variable and whether this feature was chosen as the dependent variable. There will be one regression per feature.

We will use the Bonferroni correction for all our regressions to avoid a type 1 error.

#### Qualitative Data

Qualitative analysis of the free text for the question regarding the explanation of the participants’ choice was performed, and common themes extracted. Response categories will be defined when reading the responses.

## Results

Recruitment and testing were conducted during July 2021. The deadline for the completion of the web-based questionnaire by participants was end of December 2021. We began analyzing the responses in January 2022, and the publication of results is expected at the end of 2022.

## Discussion

### Principal Findings

This study will define the preferences of functionalities of users with a specific profile (eg, what kind of functionalities are preferred by a user according to their personality). This protocol is important as its sample will enable to validate a model built on several previous studies and reviews. In turn, this will allow developers to build mobile apps that will be more efficient as adapted to each user. Thus, with this research, we will be able to better refine our conceptual framework, which will allow the mobile app designer to select features tailored to their users according to their profile and thus increase their involvement in the mHealth app.

The main interest of this research is that it gathers all the user profiles identified in the literature and all the functionalities generally implemented in mHealth. Indeed, we find studies allowing us to link personality and gamification elements [8,9,14], personality, gamer profile, and gamification elements [11], personality and sensitivity to persuasion strategies [10,27], or personality and need for cognition [28]. Moreover, these studies are not necessarily specific to the field of mobile apps for behavior change. Some studies are more focused on preferences related to video games [9,14,29] and others on the type of messages and feedback [19,30]. Therefore, our research allows to combine what has been done previously in different studies and to corroborate their findings for mHealth apps regarding user preferences according to their specific profile.

### Limitations

Our study has some limitations. We designed it to be as neutral as possible to limit preferences linked to the design of one of the prototyped functionalities. However, it is still possible that participants may prefer a certain functionality because they found it more visually attractive. Our results are also possibly not generalizable to the whole population. Indeed, since recruitment was conducted at the university and on social networks, it is expected that most participants were students aged 18-25 years. Finally, as the questionnaire was in French language and only individuals living in the canton of Geneva and the surrounding area were included, it can only be generalized to this population (ie, French-speaking people of Switzerland and France).

### Conclusion

It is important to help people adopt better health behaviors. Mobile apps are an interesting channel to support this effort because they integrate functionalities such as goal setting or self-monitoring that have been proven to foster behavior change. However, app efficiency can be improved by responding to user preferences according to their specific profiles. Our study will provide an additional evidence base to propose an accurate personalization conceptual framework for the development of future mHealth apps.

## Appendix

Multimedia Appendix 1 Presentation of the functionalities selected for our conceptual framework with their definitions and description of the screenshot.

Multimedia Appendix 2 Print version of the web-based questionnaire.

## Abbreviations

mHealth: mobile health

## Data Availability

The data sets generated during this study are available from the corresponding author upon reasonable request.
