# Supplementary material for: Personalization of Mobile Apps for Health Behavior Change: Protocol for a Cross-sectional Study
Source: JMIR Res Protoc. 2023 Jan 5;12:e38603. doi: 10.2196/38603 (PMC9853334; doi:10.2196/38603)
Supplement: Multimedia Appendix 1 [file resprot_v12i1e38603_app1.pdf]

Appendix 1

Presentation of the functionalities selected for our model with their definitions and description of the screenshot

Functionalities screenshot	Definitions
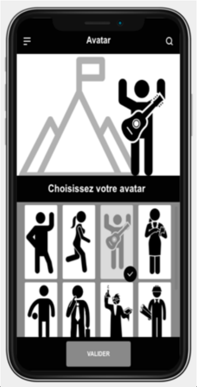	**Avatar**<br>Allows you to choose a fictitious avatar representing you in the application.
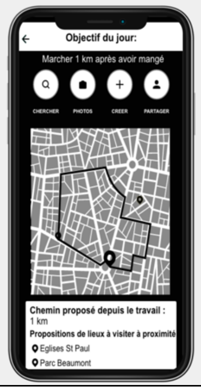	**Challenge**<br>Gives you a task to do whose difficulty depends on your skills, abilities, motivation and knowledge.
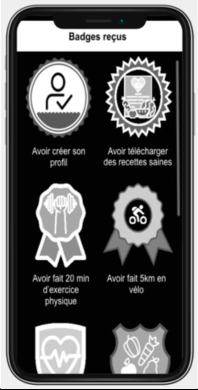	**Collectibles**<br>Allows you to collect virtual objects. In this example it is a badge.
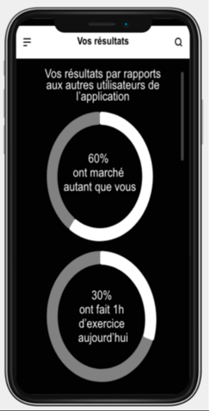	**Social comparison**<br>Allows you to compare your performance with other participants. This implies that there are no losers or winners and no direct interaction with others.

This is a Multimedia Appendix to a full manuscript published in the J Med Internet Res. For full copyright and citation information see http://dx.doi.org/10.2196/jmir.38603

<table>
<tr><td>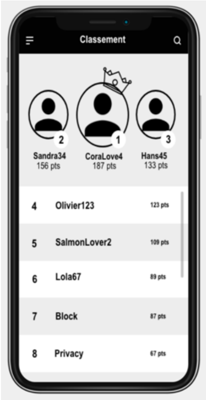
</td><td>Compétition
Allows you to compete with each other on your performance.</td></tr>
<tr><td>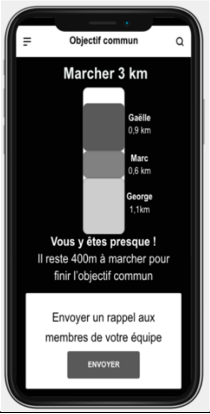
</td><td>Competitive Cooperation
Allows you to work together to achieve a common goal while competing with others.</td></tr>
<tr><td>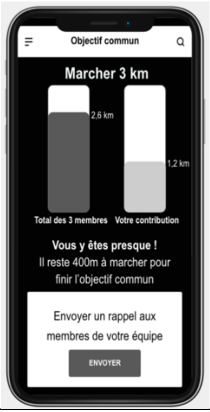
</td><td>Cooperation
Allows you to work together to achieve a common goal</td></tr>
<tr><td>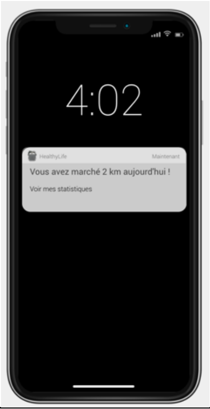
</td><td>Descriptive feedback
Provide you with feedback on your behavior.</td></tr>
</table>

This is a Multimedia Appendix to a full manuscript published in the J Med Internet Res. For full copyright and citation information see http://dx.doi.org/10.2196/jmir.38603

<table>
<tr>
<td>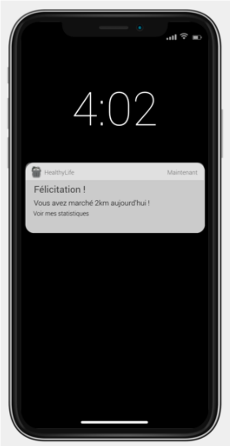
</td>
<td>Evaluative feedback
Gives you feedback with an interpretation or judgment of your behavior.</td>
</tr>
<tr>
<td>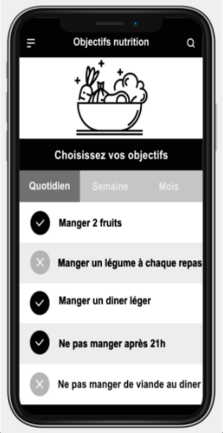
</td>
<td>Goal setting
Allows you to set your own goals.</td>
</tr>
<tr>
<td>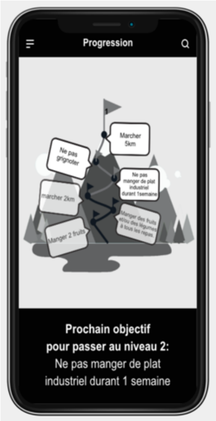
</td>
<td>Level and progress
Shows you your progress to the next level.</td>
</tr>
<tr>
<td>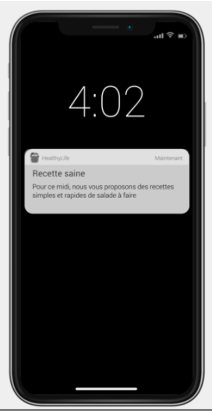
</td>
<td>Reminder
Sends you reminders via notifications</td>
</tr>
</table>

This is a Multimedia Appendix to a full manuscript published in the J Med Internet Res. For full copyright and citation information see http://dx.doi.org/10.2196/jmir.38603

<table>
<tr>
<td>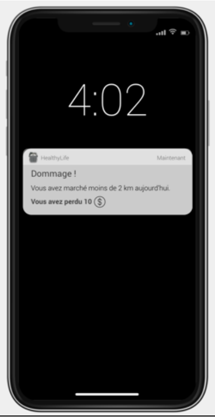
</td>
<td>Punishment
Punishes you with virtual malus</td>
</tr>
<tr>
<td>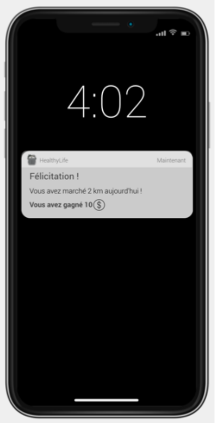
</td>
<td>Reward
Gives you virtual or real rewards.</td>
</tr>
<tr>
<td>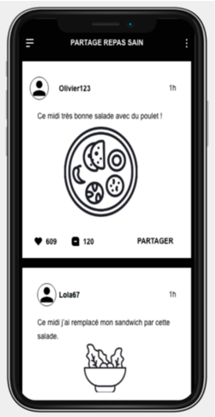
</td>
<td>Social network
Integration of a mode allowing you to exchange with each other, post messages, images etc.</td>
</tr>
<tr>
<td>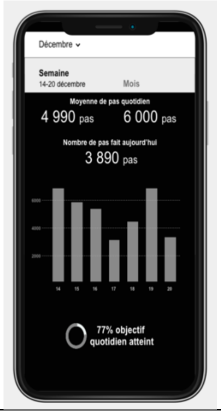
</td>
<td>Self-monitoring
Allows you to observe your behavior, the system records and presents you in a readable way (with graphs for example) your target behavior, for example the number of steps taken.</td>
</tr>
</table>

This is a Multimedia Appendix to a full manuscript published in the J Med Internet Res. For full copyright and citation information see http://dx.doi.org/10.2196/jmir.38603

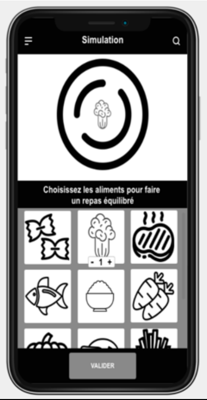	**Simulation**<br>Fictional representation of a behavior. The goal is to see the consequences of a behavior.

This is a Multimedia Appendix to a full manuscript published in the J Med Internet Res. For full copyright and citation information see http://dx.doi.org/10.2196/jmir.38603
